# Effects of Photomodulation Therapy for Delayed Onset Muscle Soreness: A Systematic Review and Meta-Analysis

**DOI:** 10.3390/jfmk10030277

**Published:** 2025-07-17

**Authors:** Yung-An Tsou, Nai-Jen Chang, Wen-Dien Chang

**Affiliations:** 1 Department of Otolaryngology-Head and Neck Surgery, China Medical University Hospital, Taichung 404327, Taiwan; d22052121@gmail.com; 2 Department of Sports Medicine, Kaohsiung Medical University, Kaohsiung 807378, Taiwan; njchang@kmu.edu.tw; 3 PhD Program in Biomedical Engineering, Kaohsiung Medical University, Kaohsiung 807378, Taiwan; 4 Department of Medical Research, Kaohsiung Medical University Hospital, Kaohsiung 807378, Taiwan; 5 Precision Sports Medicine and Health Promotion Center, Kaohsiung Medical University, Kaohsiung 807378, Taiwan; 6 Department of Sport Performance, National Taiwan University of Sport, Taichung 404401, Taiwan

**Keywords:** photomodulation therapy, delayed onset muscle soreness, visual analog scale

## Abstract

**Objectives:** This study aimed to evaluate the effects of photomodulation therapy (PMT) on delayed onset muscle soreness (DOMS). **Methods:** Controlled studies investigating PMT for DOMS were identified through systematic searches of PubMed and EMBASE databases. Selected articles were reviewed for the effects of PMT, and the outcome data were extracted according to specific assessments and time points for meta-analysis. **Results:** A total of 14 studies met the inclusion criteria, all of which evaluated the effects of PMT following the induction of DOMS. The wavelength of PMT ranged from 660 to 950 nm and was applied to one to six points on the affected muscles. Four studies provided sufficient data for quantitative synthesis, comparing PMT with the placebo in terms of visual analog scale (VAS) scores and muscle strength at 24, 48, 72, and 96 h after the induction of DOMS. The results demonstrated a statistically significant reduction in VAS scores at 72 h (pooled SMD = −0.55) and 96 h (pooled SMD = −0.56), indicating a moderate effect. Muscle strength showed significant improvement at 24 h (pooled SMD = 0.97) and 48 h (pooled SMD = 0.99), reflecting a large effect size. **Conclusions:** These findings suggested that PMT may be an effective intervention for managing DOMS, with potential effects on reducing pain, enhancing muscle strength, and decreasing biochemical markers of muscle damage.

## 1. Introduction

Photomodulation therapy (PMT) is phototherapy that utilizes non-ionizing forms of low-level laser to stimulate and expedite the healing of damaged or injured tissues [1]. This physical agent utilizes specific wavelengths of light capable of penetrating the skin to activate cellular functions, thereby enhancing blood circulation, reducing inflammation, and promoting tissue regeneration [2]. PMT has been applied in the management of a variety of conditions, including pain, inflammation, and wound healing [3]. It is typically delivered using a handheld device designed to emit targeted wavelengths of low-level laser to the affected area. Although the precise biological mechanisms underlying PMT remain incompletely understood, some evidence suggests that this facilitates the production of adenosine triphosphate (ATP), thereby providing energy for essential cellular processes involved in healing [2,4]. Additionally, it may activate specific intracellular signaling pathways and enhance microcirculation, contributing to reduced inflammation and accelerating tissue repair [1,3]. Overall, PMT is regarded as a safe, non-invasive modality with minimal reported adverse effects.

Delayed onset muscle soreness (DOMS) is a well-characterized physiological phenomenon, typically presenting 24 to 72 h following unaccustomed or strenuous physical exertion. It is marked by symptoms such as muscle pain, stiffness, and tenderness [5]. While DOMS is frequently associated with resistance training, it may also result from endurance-based or eccentric exercises, including running, hiking, and plyometric training [5,6]. Although the precise pathophysiological mechanisms underlying DOMS remain incompletely understood, it is widely hypothesized to involve microtrauma to muscle fibers and connective tissues, subsequently triggering a localized inflammatory response [7]. Clinically, affected individuals may exhibit muscle soreness, reduced range of motion in joints, and localized swelling [8]. Despite DOMS decreasing spontaneously within a few days, various interventions, such as cryotherapy, low-level laser therapy, vibration therapy, ultrasound, massage, and stretching exercise, have been investigated to alleviate the uncomfortable symptoms [9]. PBM is a non-invasive therapy using low-level laser therapy to reduce inflammation, relieve pain, and promote tissue healing. Among these, it has garnered increasing interest for its potential to attenuate muscle soreness and facilitate recovery. Further evidence-based research is warranted for the application of PMT, and for elucidating the underlying positive effects in DOMS.

Several physical modalities, including massage and physical therapy, have been used to alleviate the symptoms of DOMS and facilitate fatigued muscle recovery [10]. Nonetheless, the application of PMT, particularly low-level laser, for the management of DOMS remains an emerging area of investigation. Preliminary studies have indicated positive outcomes. A previous review examined the effects of PMT on enhancing muscular performance and reducing muscular fatigue within an exercise protocol [11]. The meta-analysis of time to exhaustion, number of repetitions, isometric peak torque, and blood lactate levels of the muscles demonstrated a low to moderate quality of evidence, with beneficial effects in favor of PMT [11]. However, the study did not include a comparative analysis of the effects on DOMS. Another review examined whether PMT modulated chemical mediators involved in the inflammatory response, specifically blood lactate (BL) and creatine kinase (CK) [12]. The findings demonstrated that PBT significantly reduced serum levels of BL and CK when applied post-exercise. Nonetheless, due to the limited number of studies included, additional review articles are needed to further substantiate these findings. Hence, the current review study is limited in scope, with relatively few high-quality controlled trials and systematic reviews evaluating the effects of PMT in this specific context. As such, further methodologically rigorous studies are warranted to elucidate the underlying mechanisms and to establish evidence-based effects for its use. The aim of this study was to conduct a systematic review and meta-analysis to evaluate the effects of PMT for DOMS.

## 2. Methods

### 2.1. Search Strategy and Data Sources

In accordance with the Preferred Reporting Items for Systematic Reviews and Meta-Analyses (PRISMA) guidelines, a comprehensive literature search was conducted using the PubMed and EMBASE electronic databases. The search strategy employed combinations of the following keywords, i.e., “low level laser”, “photomodulation”, and “delayed onset muscle soreness”. The term “[(delayed onset muscle soreness) AND (low level laser) OR (photomodulation)]” was used to search comprehensive and relevant results. The initial search encompassed all results published between 1996 and 2025. Articles were included if they met the following eligibility criteria: (1) participants who have undergone an intervention designed to induce DOMS; (2) participants in whom photomodulation therapy has been applied as a treatment modality for the affected muscles; and (3) studies that included a placebo group or a control group for comparison. The exclusion criteria were as follows: (1) retrospective studies or case reports; (2) non-English language publications; and (3) articles without full-text availability. Two researchers screened the titles and abstracts of the retrieved records to identify eligible studies. Full texts of potentially relevant articles were then obtained and reviewed. All included articles were compiled into a summary table, and data were extracted for meta-analysis.

### 2.2. Data Extraction and Analysis

The data extracted from the included studies were coded and analyzed in three parts. The first part involved the descriptive characteristics of each study, including author and year, experimental design, sample size, targeted DOMS muscle groups, and study quality. Study quality was assessed using the Physiotherapy Evidence Database (PEDro) scale, which evaluates the risk of bias and applicability. The PEDro scale consists of 11 items, with the first item not scored. The total score ranges from 0 to 10, with higher scores indicating better methodological quality [13]. Study quality assessment was conducted independently by two researchers. The second part documented the parameters of PTM used in each study, along with the outcome measures, assessment time points, and results. These were organized and synthesized accordingly. The third part involved data extraction and meta-analysis. To minimize psychological effects, only the PTM and placebo groups were included in the analysis. Outcome data were extracted from each study based on the specific assessment indicators and time points, and were subjected to meta-analysis. The adequacy of the outcome measures across studies was reviewed to allow for the evaluation of the effectiveness of different variables at distinct assessment time points.

### 2.3. Statistical Analysis

Meta-analyses were conducted using MedCalc version 14 (MedCalc Software, Oostende, Belgium). The standardized mean difference (SMD) with a 95% confidence interval (CI) was used to analyze the effect size (ES) of each included study. To be eligible for inclusion in the meta-analysis, studies were required to report both means and standard deviations. The ES was calculated for each outcome measure at multiple time points, such as immediately, 24, 48, 72, and 96 h after the induction of DOMS. Statistical heterogeneity was assessed using the I^2^ statistic to determine the appropriateness of applying either a fixed-effects or random-effects model. The magnitude of effect sizes was classified according to Cohen’s criteria, with small (0.2 ≤ ES < 0.5), moderate (0.5 ≤ ES < 0.8), and large (0.8 ≤ ES) [14]. A *p*  <  0.05 was considered statistically significant.

## 3. Results

**Figure 1:**
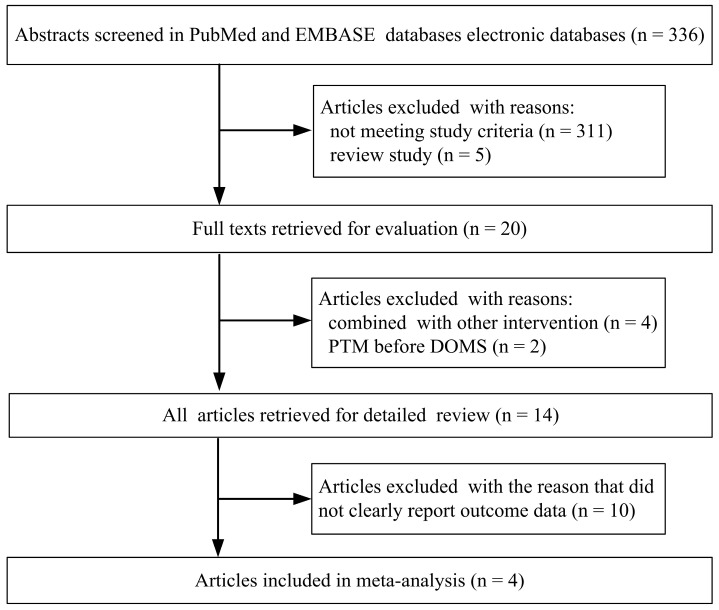
Study flow diagram.

A total of 336 articles were initially identified through electronic database searches (Figure 1). Three hundred and sixteen articles were excluded as they did not meet the study criteria or were review studies, leaving 20 full-text articles for detailed assessment. Upon further evaluation, four studies were excluded for combining PTM with other interventions, and two studies were excluded for examining the effects of PTM administered prior to the onset of DOMS. Ultimately, 14 articles were deemed appropriate based on the eligibility criteria and were selected by two independent reviewers [15,16,17,18,19,20,21,22,23,24,25,26,27,28]. Of these, 10 articles did not report outcome data with sufficient clarity, resulting in the inclusion of four articles in the final meta-analysis. Due to substantial variability in outcome assessments across studies, only visual analogue scale (VAS) scores and muscle strength measurements were analyzed immediately and at 24, 48, 72, and 96 h after the induction of DOMS [18,19,20,22].

As shown in Table 1, 10 of the included articles employed a parallel study design [15,16,18,19,20,21,22,23,24,26,27,28], while 2 utilized a crossover design [17,25]. Five studies applied PTM to the lower extremities (i.e., quadriceps, hamstrings, and calf muscles) [15,16,18,22,25], whereas nine studies focused on the upper extremities (i.e., biceps brachii) [17,19,20,21,23,24,26,27,28]. All studies compared PTM with a placebo group, except for one study that used a control group for comparison [15]. The methodological quality of the studies, as assessed via PEDro scale, ranged from 4 to 9 points.

The PTM parameters related to DOMS are presented in Table 2. The wavelength of PTM ranged from 660 to 950 nm and was applied to 1 to 6 points on the affected muscles [15,16,17,18,19,20,21,22,23,24,25,26,27,28]. In the studies by Chang et al. [19] and Fleckenstein et al. [21], acupuncture points on DOMS muscle were selected as the sites of irradiation. As shown in Table 3, muscle soreness and pain associated with DOMS were assessed using the visual analogue scale (VAS), the McGill pain questionnaire, and pain pressure threshold (PPT) measurements [15,16,17,18,19,20,21,22,24,26,27,28]. The muscle strength and swelling condition of DOMS muscles were evaluated using limb circumference, range of motion (ROM), muscle strength tests, rating of perceived exertion (RPE), and force sense assessments [15,17,18,19,20,21,22,23,24,26,27,28]. Biochemical markers of muscle damage were examined through assays such as CK, 2,4-dinitrophenylhydrazine (DNPH), thiobarbituric acid reactive substances (TBARSs), BL, and C-reactive protein [20,22,23,25]. Two studies utilized performance-based tests, including the single-leg forward jump, vertical jump, and the agility T-test, to assess muscular performance [15,16]. All studies evaluated outcomes both before and after the PTM intervention, as well as during follow-up assessments. Among the included studies, four reported no significant effects in the PTM group [15,21,27,28], while ten reported positive effects following PTM application [16,17,18,19,20,22,23,24,25,26].

**Figure 2:**
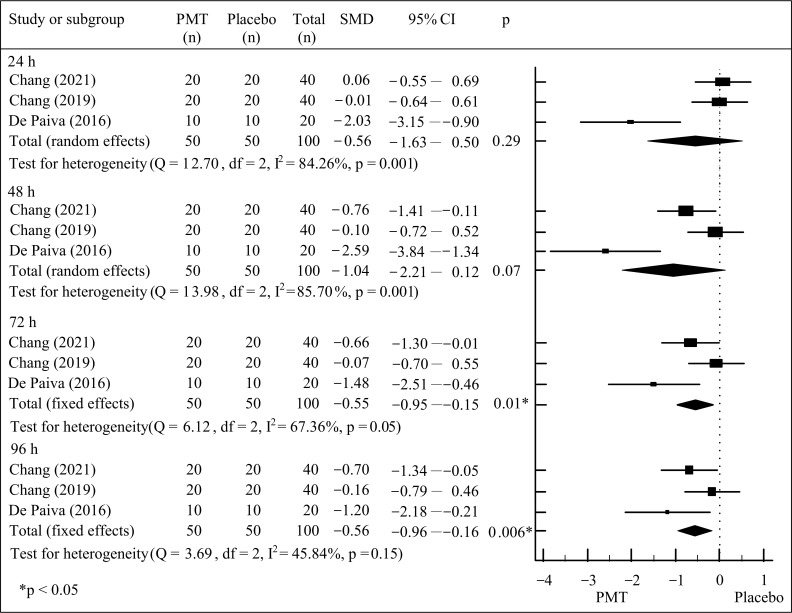
Meta-analysis of VAS between PMT and placebo groups [18,19,22].

VAS and muscle strength assessments were reported immediately and at 24, 48, 72, and 96 h after the induction of DOMS, providing sufficient data for meta-analysis [18,19,20,22]. When comparing PMT with the placebo group, the meta-analytic results revealed statistically significant reductions in VAS at 72 h (pooled SMD = −0.55; 95% CI: −0.95 to −0.15; *p* < 0.05) and 96 h (pooled SMD = −0.56; 95% CI: −0.96 to −0.16; *p* < 0.05) after the induction of DOMS (Figure 2). Between-study heterogeneity, with I^2^ values of 67.36% (72 h post-intervention, *p* < 0.05) and 45.84% (96 h post-intervention, *p* < 0.05), was noted. These findings suggest that PMT produces a moderate effect in reducing perceived muscle soreness at 72 and 96 h after the induction of DOMS.

**Figure 3:**
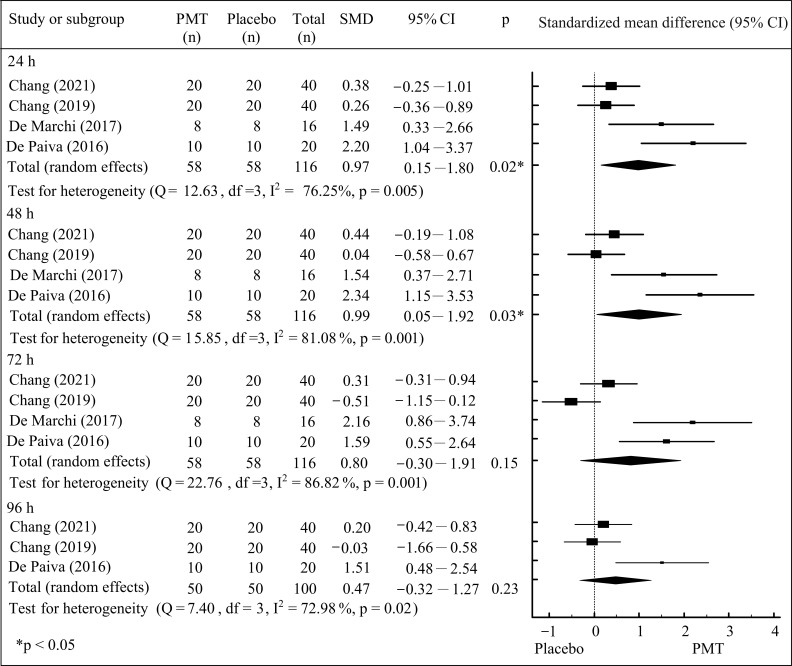
Meta-analysis of muscle strength between PMT and placebo groups [18,19,20,22].

Compared to the placebo group, the results of the meta-analyses demonstrated that PMT led to statistically significant improvements in muscle strength at 24 h (pooled SMD = 0.97; 95% CI: 0.15 to 1.80; *p* < 0.05) and 48 h (pooled SMD = 0.99; 95% CI: 0.05 to 1.92; *p* < 0.05) after the induction of DOMS (Figure 3). The between-study heterogeneity was I^2^ values of 76.25% at 24 h and 81.08% at 48 h after the induction of DOMS (both *p* < 0.05). These findings suggested that PMT may facilitate the recovery of muscle strength at 24 and 48 h after the induction of DOMS, and were considered to represent a large effect.

## 4. Discussion

PMT may be a potential modality for attenuating DOMS and promoting muscle recovery. This systematic review and meta-analysis synthesized and evaluated existing controlled trials investigating the effects of PMT on muscle pain, muscle strength, biochemical markers of muscle damage and muscular performance. Despite some variability in outcomes, the overall findings revealed that PMT had positive effects, particularly in the decrease in VAS and increase in muscle strength recovery.

Biochemical markers provide critical evidence regarding the efficacy of PMT in modulating post-exercise muscle damage. Some studies have consistently demonstrated that PMT leads to reductions in key biomarkers such as CK, DNPH, and TBARS [20,22,23,25]. De Marchi et al. reported significant decreases in CK, TBARS, and DNPH levels after PMT compared to the placebo, suggesting a protective effect against muscle membrane damage and oxidative stress [20]. Felismino et al. and de Paiva et al. observed lower CK levels in subjects treated with PMT, reinforcing the therapy’s role in mitigating muscle injury [22,23]. The reduction in oxidative stress markers such as TBARS and DNPH is of particular importance. These markers indicate lipid peroxidation and protein oxidation, both of which are increased following strenuous exercise [29]. PMT is thought to boost the activity of endogenous antioxidant enzymes like superoxide dismutase and catalase, thereby neutralizing reactive oxygen species [20]. Furthermore, Leal Junior et al. reported decreased BL levels after PMT, which may reflect improved mitochondrial efficiency and metabolic clearance [25].

Pain relief is the most consistently assessed benefit of PMT in the reviewed studies. Chang et al. demonstrated significant improvements in PPT and ROM in PMT-treated individuals relative to the placebo [18]. Similarly, another study reported within-group reductions in VAS scores and improvements in PPT after PMT, although between-group differences were primarily noted in limb circumference [19]. Douris et al. also found that PMT alleviated subjective pain, as evidenced by decreased scores on the VAS and the McGill Pain Questionnaire [26]. D’Amico et al. identified localized pain reduction in the calf muscle, suggesting the potential for muscle-specific benefits [16]. Conversely, some studies observed no significant differences between the PMT and control groups, highlighting that treatment outcomes may depend on PMT parameters [17,27,28]. Our meta-analytic results revealed statistically significant reductions in VAS at 72 h (pooled SMD = −0.55) and 96 h (pooled SMD = −0.56) after the induction of DOMS, and that it had a moderate effect. The analgesic effects of PMT may occur through some biological mechanisms. Primarily, PMT enhances mitochondrial activity by stimulating cytochrome c oxidase, which increases ATP synthesis and supports cellular repair [23]. In addition, PMT appears to reduce oxidative stress and suppress pro-inflammatory cytokines, such as TNF-α and IL-6, thus lowering nociceptor sensitization [20,25]. Enhanced local vasodilation from increased nitric oxide production further improves microcirculation and facilitates the removal of inflammatory metabolites [4,26]. Although these processes collectively contribute to pain relief, the variation in protocols (i.e., differences in wavelength, intensity, and treatment duration of PMT) across studies likely accounts for the discrepancies in clinical outcomes.

PMT may expedite the restoration of muscle strength following eccentric exercise. Our meta-analytic results revealed that PMT led to improvements in muscle strength at 24 h (pooled SMD = 0.97) and 48 h (pooled SMD = 0.99) after the induction of DOMS, and had a large effect. Some studied demonstrated PMT significantly improved muscle strength compared to those in placebo groups [18,19,22,24]. These improvements were often accompanied by reductions in biomarkers of muscle damage, such as CK and BL, suggesting that PMT not only alleviates symptoms but may also promote underlying muscle repair [3,30]. Douris et al. similarly observed positive effects on strength following PMT [26]. At the cellular level, PMT is believed to facilitate muscle recovery by upregulating mitochondrial respiration and ATP production, which are essential for the repair of contractile proteins and cellular membranes [23,31]. Additionally, by downregulating inflammatory mediators such as TNF-α, PMT helps preserve the structural integrity of muscle fibers [20,32,33]. There is also evidence suggesting that PMT may stimulate satellite cell activation, thereby promoting myogenic differentiation and the regeneration of muscle fibers [22]. Malta et al. and D’Amico found that PMT did not accelerate muscular performance recovery in single-leg forward jump, vertical jump, and agility tests following DOMS [15,16]. In contrast, a prior study has demonstrated the improved recovery of maximal voluntary contraction, which represents a high-force, low-velocity task. The single-leg forward jump, vertical jump agility T-test are categorized as low-force, high-velocity activities that are more constrained by neurological factors [34]. This suggested that PMT may be less effective for recovering short, explosive movements that rely heavily on neurological input compared to longer-duration activities where fatigue is more prominent [16].

The current study had some limitations. First, the variability in PMT parameters and assessments for DOMS complicates direct comparisons among the studies. Second, insufficient data regarding outcomes may have impacted the results of the meta-analysis. Although our results indicated that PMT demonstrated moderate to large effect sizes for alleviating muscle soreness and enhancing muscle strength following DOMS, further studies are still required to validate and support these findings.

In the current study, a systematic review was conducted to evaluate the effects of PMT on DOMS and to compare outcomes following PMT intervention. Compared to the placebo group, PMT was associated with a reduction in muscle pain and an enhancement in muscle strength recovery after DOMS. These findings may have practical implications for sports physical therapy, particularly in managing DOMS-related symptoms. However, further more high-quality studies with larger sample sizes and well-defined PMT parameters are necessary to strengthen the evidence base and increase confidence in the estimated effects. While the present study focused on the post-exercise application of PMT, future research should also explore its potential as a preventive intervention when applied prior to exercise to mitigate the onset of DOMS. Furthermore, the meta-analysis of only four articles was feasible for evaluating the changes in pain and muscle strength following DOMS, and some evidence has indicated that PBT may be effective for these outcomes. More studies are also needed to determine the effect of PMT on the improvement in outcomes related to DOMS. We suggest strengthening the evidence regarding the effects of PMT for DOMS to enable a meta-analysis supported by sufficient and diverse outcome measures.

## 5. Conclusions

PMT may serve as an effective intervention for managing DOMS. Several included articles demonstrated its potential ability to reduce pain, enhance muscle strength, and lower biochemical markers indicative of muscle damage. Despite the presence of heterogeneity among studies, it was found that PMT had moderate to large effects on alleviating muscle soreness and promoting the recovery of muscle strength following the induction of DOMS.

## Figures and Tables

**Table 1:** Characteristics of study design, DOMS muscle, and article quality.

Author (Years)	Study Design	DOMS Muscle	Group (Sample Size, n)	Quality
Ma (2022) [15]	Parallel	Quadriceps	PMT (n = 12) Control (n = 15)	6
D’Amico (2022) [16]	Parallel	Quadriceps, hamstring and calf	PMT (n = 16) Placebo (n = 17)	7
Azuma (2021) [17]	Crossover	Biceps brachii	PMT (n = 15) Placebo (n = 15)	7
Chang (2021) [18]	Parallel	Quadriceps	PMT (n = 20) Placebo (n = 20)	6
Chang (2019) [19]	Parallel	Biceps brachii	PMT (n = 20) Placebo (n = 20)	5
De Marchi (2017) [20]	Parallel	Biceps brachii	PMT (n = 8) Placebo (n = 8)	9
Fleckenstein(2016) [21]	Parallel	Biceps brachii	PMT (n = 12) Placebo (n = 12) Control (n = 12)	7
De Paiva (2016) [22]	Parallel	Quadriceps	PMT (n = 10) Placebo (n = 10)	9
Felismino(2014) [23]	Parallel	Biceps brachii	PMT (n = 11) Placebo (n = 11)	6
Borges (2014) [24]	Parallel	Biceps brachii	PMT (n = 8) Placebo (n = 9)	5
Leal Junior (2011) [25]	Crossover	Hamstring and calf	PMT (n = 6) Placebo (n = 6)	5
Douris (2006) [26]	Parallel	Biceps brachii	PMT (n = 9) Placebo (n = 9) Control (n = 9)	4
Craig (1999) [27]	Parallel	Biceps brachii	PMT (n = 12) Placebo (n = 12) Control (n = 12)	4
Craig (1996) [28]	Parallel	Biceps brachii	PMT (n = 12) Placebo (n = 12) Control (n = 12)	4

DOMS, delayed onset muscle soreness; PMT, photomodulation therapy.

**Table 2:** Characteristics of PMT parameters on DOMS.

Author (Years)	Wavelength (Nm)	Frequency (Hz)	Output (mW)	PMT Intervention
Ma (2022) [15]	810 ± 30	Continuous	400	Number of points: 6 points of quadriceps, 2 points of hamstring and 2 points of calf muscle; time per point: 125 s; 1 section per day
D’Amico (2022) [16]	650	Continuous	200	Number of points: 6 points of quadriceps muscle; time per point: 30 s
Azuma (2021) [17]	808	Continuous	100	Number of points: 4 points of biceps muscle; time per point: 70 s
Chang (2021) [18]	830	10	210	Number of points: 6 points of quadriceps muscle; time per point 10 min
Chang (2019) [19]	830	10	60	Number of points: 2 points of biceps muscle; time per point 10 min
De Marchi (2017) [20]	660/850	Continuous	10/30	Number of points: 1 points of biceps muscle; time per point: 30 s
Fleckenstein (2016) [21]	NA	NA	NA	Number of points: 8 points of biceps muscle
De Paiva (2016) [22]	905/875	1000/16	15/70	Number of points: 6 points of quadriceps muscle; time per point 300 s
Felismino (2014) [23]	808	Continuous	100	Number of points: 4 points of biceps muscle; time per point: 10 s
Borges (2014) [24]	630	Continuous	300	Number of points: 4 points of biceps muscle; time per point: 30 s
Leal Junior (2011) [25]	660/835	Continuous	10	Number of points: 4 points of bilateral hamstring muscle and 1 point of calf muscle; time per point: 30 s
Douris (2006) [26]	660/880	NA	NA	Number of points: 2 points of biceps muscle; time per point: 80 s
Craig (1999) [27]	660–950	73	534	Number of points: NA; time per point: 4 min on biceps muscle
Craig (1996) [28]	660–950	2.5, 5 or 20	NA	Number of points: NA; time per point: 12 min on biceps muscle

PMT, photomodulation therapy; NA, not available.

**Table 3:** Characteristics of assessments, assessments times and outcomes for DOMS.

Author (Years)	Assessments	Time Point	Outcomes
Ma (2022) [15]	VAS, PPT, muscle strength, single-leg forward jump	Before, 24 h, 48 h, 72 h and 96 h	No significant differences in all assessments within and between groups
D’Amico (2022) [16]	VAS, vertical jump, agility T-test	Before, immediately, 24 h, 48 h, 72 h and 96 h	A decrease of VAS on calf muscle between groups * No significant differences in vertical jump, and agility T-test
Azuma (2021) [17]	VAS, RPE	Before, immediately, 24 h, 48 h, and 72 h	An increase of RPE in PMT group * No significant differences in VAS and PRE between groups
Chang (2021) [18]	VAS, PPT, limb circumference, ROM, muscle strength	Before, immediately, 24 h, 48 h, 72 h, and 96 h	Significant improvements on PPT and ROM between groups * No significant differences in limb circumference, muscle strength between groups between groups.
Chang (2019) [19]	VAS, PPT, force sense, limb circumference, muscle strength	Before, immediately, 24 h, 48 h, 72 h, and 96 h	Significant changes on VAS, PPT, limb circumference, muscle strength in PMT group* Only a significant difference in limb circumference between groups *
De Marchi (2017) [20]	VAS, muscle strength, CK, TBARS, DNPH	Before, immediately, 60 min, and 24 h, 48 h and 72 h	Significant differences in CK, TBARS, and DNPH between groups *
Fleckenstein (2016) [21]	VAS, PPT, muscle strength	Before, 24, 48 and 72 h	No significant differences in VAS, PPT, muscle strength among the groups
De Paiva (2016) [22]	VAS, muscle strength, CK	Before, immediately, 1 h, 24 h, 48 h, 72 h and 96 h	Significant differences in VAS, muscle strength and CK between the groups *
Felismino (2014) [23]	RPE, muscle strength, CK	Before, immediately, 24 h, 48 h, and 72 h	A significant difference in CK between groups *
Borges (2014) [24]	VAS, ROM, muscle strength	Before, 24 h, 48 h, 72 h and 96 h	Significant differences in VAS, ROM, muscle strength between groups *
Leal Junior (2011) [25]	CK, BL, C-reactive protein	Before and immediately	Significant decreases in CK and BL in PMT group*, but no significant differences in all variables between the groups
Douris (2006) [26]	VAS, McGill pain questionnaire, limb circumference, ROM	Before, 24 h, 48 h, 72 h and 96 h	Significant differences in VAS, McGill pain questionnaire between groups *
Craig (1999) [27]	VAS, PPT, ROM	Before and 1–11 days	No significant differences in VAS, PPT and ROM among the groups
Craig (1996) [28]	VAS, McGill pain questionnaire, PPT, ROM,	Before, 24 h, 48 h, and 72 h	No significant differences in VAS, McGill pain questionnaire, PPT and ROM among the groups

* *p* < 0.05. VAS, visual analogue scale; PPT, pain pressure threshold; PMT, photomodulation therapy; RPE, rating of perceived exertion; ROM, range of motion; CK, creatine kinase; TBARS, thiobarbituric acid reactive substances; DNPH, 2,4-dinitrophenylhydrazine.
